# Prognostic value of lactate levels and lactate clearance in sepsis and septic shock with initial hyperlactatemia: A retrospective cohort study according to the Sepsis-3 definitions

**DOI:** 10.1097/MD.0000000000024835

**Published:** 2021-02-19

**Authors:** Seong Geun Lee, Juhyun Song, Dae Won Park, Sungwoo Moon, Han-jin Cho, Joo Yeong Kim, Jonghak Park, Jae Hyung Cha

**Affiliations:** a Department of Emergency Medicine; b Division of Infectious Diseases, Department of Internal Medicine; c Medical Science Research Center, Korea University Ansan Hospital, Ansan, Republic of Korea.

**Keywords:** lactate, lactate clearance, mortality, sepsis, septic shock

## Abstract

The 2016 Surviving Sepsis Campaign guidelines suggest guiding resuscitation to normalize lactate levels in patients with sepsis-associated hyperlactatemia as a marker of tissue hypoperfusion. This study evaluated the prognostic value of lactate levels and lactate clearance for 30-day mortality in patients with sepsis and septic shock diagnosed in the emergency department.

We performed a retrospective cohort study of sepsis patients with initial lactate levels of ≥2 mmol/L. All patients met the Sepsis-3 definitions. The prognostic value of 6-hour lactate levels, 6-hour lactate clearance, 6-hour lactate metrics (≥2 mmol/L), and lactate clearance metrics (<10%, <20%, and <30%) was evaluated. We compared the sensitivity and specificity between metrics.

Of the 363 sepsis and septic shock patients, 148 died (30-day mortality: 40.8%). Nonsurvivors had significantly higher 6-hour lactate levels and lower 6-hour lactate clearance than those of survivors. Six-hour lactate levels and 6-hour lactate clearance were associated with 30-day mortality after adjusting for potential confounders (odds ratio, 1.191 [95% confidence interval (CI), 1.097–1.294] and 0.989 [0.983–0.995], respectively). Six-hour lactate levels had better prognostic value than 6-hour lactate clearance (area under the curve, 0.720 [95% CI, 0.670–0.765] vs 0.656 [0.605–0.705]; *P* = .02). Six-hour lactate levels of ≥3.5 mmol/L and 6-hour lactate clearance of <24.4% were the optimal cut-off value in predicting the 30-day mortality. The prognostic value of 6-hour lactate metrics and 6-hour lactate clearance metrics did not differ. Six-hour lactate levels (≥2 mmol/L) had the highest sensitivity (89.2%).

Six-hour lactate levels proved to be more accurate in predicting 30-day mortality than 6-hour lactate clearance and initial lactate levels.

## 1 Introduction

Lactate is known to play a major role in energy production and cellular metabolism.^[1]^ Lactic acidosis can occur in various conditions such as sepsis, liver diseases, trauma, shock, vigorous exercises, drug intoxication, and cancer.^[2]^ The 2016 Surviving Sepsis Campaign guidelines suggest guiding resuscitation to normalize lactate levels in patients with sepsis-associated hyperlactatemia as a marker of tissue hypoperfusion (hypoxia or oxygen debt).^[3,4]^ Previous studies showed that other nonhypoxic causes can contribute to the elevation of lactate levels.^[5–10]^ Besides tissue hypoxia, mitochondrial defect in oxygen utilization, impaired function of pyruvate dehydrogenase, mismatch between oxygen delivery and oxygen consumption, and accelerated aerobic glycolysis driven by sepsis-associated inflammation have been proposed as potential causes of lactate elevation.

Although the physiologic source of lactate production during sepsis development remains controversial,^[11]^ several studies have reported that increased lactate levels and lactate clearance are associated with mortality in septic patients.^[8,12–16]^ However, since the release of the latest Third International Consensus Definitions for Sepsis and Septic Shock (Sepsis-3),^[17]^ there have been limited studies investigating the prognostic performance of lactate levels and lactate clearance in both sepsis and septic shock patients diagnosed according to the Sepsis-3 definitions.

In this study, we aimed to evaluate the prognostic value of lactate levels and lactate clearance in patients with sepsis and septic shock diagnosed using Sepsis-3 in the emergency department (ED). Furthermore, we aimed to compare the performance of various lactate metrics in predicting mortality.

## 2 Materials and methods

### 2.1 Study design and population

This study was performed at the general ED of a tertiary teaching hospital with an annual census of 50,000 patients. The institutional review board at Korea University Medical Center approved the study, and the requirement to obtain informed consent was waived (IRB no. 2019AS0274). This study was conducted in accordance with the Declaration of Helsinki.

From January 2016 to December 2019, we enrolled adult patients (≥19 years old) who were diagnosed with sepsis and septic shock in the ED using an Intelligent Sepsis Management System (i-SMS). The intelligent sepsis management system (i-SMS) is a computerized program (quick Sequential Organ Failure Assessment [qSOFA] alert system) that was developed at our institution to facilitate the early detection and appropriate management of sepsis in accordance with the Sepsis-3 definitions and the 2016 Surviving Sepsis Campaign (SSC) guidelines.^[18]^ The i-SMS is activated according to the initial registration information including vital signs, mental status, reasons for visit (medical or traumatic), epidemiological data and do-not-resuscitate (DNR) order. Because the i-SMS is designed not to be activated in patients who visit ED for trauma care, patients with trauma, drug intoxication, drowning, vigorous exercises or animal bite are originally excluded. Additionally, the i-SMS is not activated in patients with DNR order. All patients were managed according to the SSC bundle, including the administration of broad-spectrum antibiotics, blood culture, fluid resuscitation, and initial lactate measurement.^[3,4]^ During the study period, patients who were diagnosed with sepsis in the ED met all of the following conditions: an initial positive qSOFA result, presence of infection, and a Sequential Organ Failure Assessment (SOFA) score increase of ≥2. Finally, we excluded patients who did not undergo a repeat 6-hour lactate measurement, and those with unknown 30-day mortality and initial lactate levels of less than 2 mmol/L. Supplementary Table 1 showed baseline characteristics of excluded patients who lacked 6-hour lactate levels. We compared epidemiologic and clinical characteristics of included patients with those of excluded patients.

### 2.2 Definitions

The Sepsis-3 definitions recommend the use of the qSOFA score for sepsis screening in patients outside the intensive care unit.^[17]^ The qSOFA score uses 3 criteria, assigning 1 point for low blood pressure (systolic blood pressure ≤100 mm Hg), high respiratory rate (≥22 breaths per min), or altered mental status (Glasgow Coma Score <15). The score ranges from 0 to 3 points. A positive qSOFA score is indicated by the presence of 2 or more qSOFA points near the onset of infection. A positive qSOFA score was used as an inclusion criterion for this study. According to the Sepsis-3 definitions, the diagnostic criteria for sepsis include an increase in SOFA score by 2 points or more as a result of current infections. The criteria for septic shock include vasopressor requirement to maintain a mean arterial pressure of 65 mm Hg and serum lactate level greater than 2 mmol/L, despite adequate fluid resuscitation. Lactate clearance (%) was calculated as follows:

([Initial lactate **−** subsequent 6-hour lactate]/initial lactate) × 100.

### 2.3 Data collection

Demographic data, comorbidity, severity, and laboratory test results were obtained. The SOFA score was calculated upon initial recognition of sepsis or septic shock. In accordance with the SSC 2016 guidelines, serum lactate levels (initial and subsequent) of the enrolled patients were measured. The initial serum lactate levels were measured upon recognition of sepsis or septic shock, and the subsequent lactate levels were recorded after the initial measurement. As the subsequent lactate levels were measured at different times after the initial measurement, we only included patients who underwent a 6-hour lactate level measurement (measured between 5 and 7 hours from initial measurement). Lactate level was measured using the Cobas 8000 c702 (Roche Diagnostics System, Rotkreuz, Switzerland), an automated system for immunoassays. The predictive value of 6-hour lactate levels and lactate clearance for 30-day mortality was evaluated.

### 2.4 Statistical analysis

Based on the results of the previous study, we expected 30-day all-cause mortality to be 35%. The previous study showed that 6-hour lactate levels predicted mortality better than lactate clearance [area under the receiver operating characteristic (ROC) curves (AUC), 0.70 vs 0.65].^[16]^ Our hypothesis was that we would observe similar AUCs in our study. Assuming 90% power with a 2-sided alpha levels of 0.05, our study required 340 patients (212 survivors and 128 non-survivors).

We used descriptive statistics to summarize the study population. Continuous variables were presented as means ± standard deviations or medians with interquartile ranges (IQRs) according to the distribution (normal or not) of the data. Categorical variables were expressed as counts and percentages. The Student *t* test or Mann–Whitney *U* test was used to compare continuous variables between the survivor and nonsurvivor groups. The Chi-Squared or Fisher exact test was used to compare categorical variables.

The association between all lactate metrics and 30-day mortality was assessed using a logistic regression model. After conducting an unadjusted analysis, we constructed a multivariate model after adjusting for age, the SOFA score and initial lactate levels. The odds ratios (ORs) with their corresponding 95% confidence intervals (CIs) for each model were presented. We evaluated the performance of lactate levels and lactate clearance using the area under the receiver operating characteristic (AUROC) curves. These AUROC curves were compared using Delong method^[19]^ and Bonferroni-correction for multiple comparisons. We reported the sensitivity, specificity, positive predictive value, and negative predictive value of each metric and used the McNemar test to compare the sensitivity and specificity between the metrics.^[20]^ All of the enrolled patients were grouped according to the optimal cut-off values of 6-hour lactate level and 6-hour lactate clearance. Kaplan–Meier curve analysis and a log-rank test were performed to assess the cumulative survival probability and compare the survival curves of groups with lower lactate levels or lactate clearance with those of the groups with higher lactate levels or clearance. Kaplan–Meier curve analysis and a log-rank test were performed to assess the cumulative survival probability and compare the survival curves of sepsis without shock or no multi-organ failure with septic shock or multi-organ failure.

Subgroup analysis was conducted according to the disease severity (sepsis vs septic shock). Two sensitivity analyses were conducted to test the prognostic value of each variable. First, we conducted a sensitivity analysis excluding patients who were transferred from other hospitals. Second, we conducted a sensitivity analysis using the Sepsis-2 definitions and included severe sepsis and septic shock in accordance with the previous definitions.

All analyses in this study were performed using the MedCalc for Windows, version 19.1.6 (MedCalc Software, Mariakerke, Belgium) and SPSS version 23.0 (IBM, Armonk, NY, USA). A *P* value of less than .05 was considered to be significant.

## 3 Results

### 3.1 Baseline characteristics

**Figure 1:**
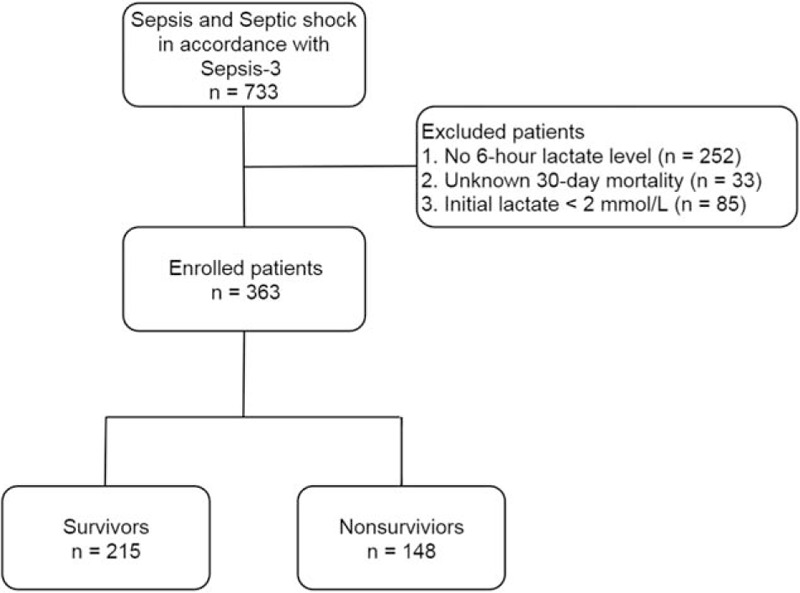
Flow chart of the study population.

From the registry collected by i-SMS, 733 patients were initially included in this study. All patients met the diagnostic criteria of sepsis or septic shock in accordance with the Sepsis-3 definitions. We excluded 252 patients without subsequent 6-hour lactate levels, 33 with unknown outcomes to predict the 30-day mortality, and 85 with initial lactate levels of less than 2 mmol/L. Therefore, a final cohort of 363 patients was included in the analysis (Fig. 1). Of the total patients, 215 survived and 148 died, resulting in a 30-day mortality rate of 40.8%.

The baseline characteristics of the study population are presented in Table 1. The median age (IQR) was 76 (64–82) years, and 204 patients (56.2%) were men. The subsequent 6-hour lactate levels in nonsurvivors were higher than those in survivors (4.6 mmol/L [IQR, 2.7–7.1] vs 2.3 mmol/L [IQR, 1.7–3.7]; *P* = .00). Lactate clearance in nonsurvivors was lower than that in survivors (15.7% [IQR, –6.7%–36.6%] vs 33.3% [IQR, 11.1%–51.6%]; *P* = .00).

**Table 1:** Baseline characteristics of the study population.

Variable	Total (n = 363)	Survivors (n = 215)	Non-survivors (n = 148)	*P* value
Age, median (IQR)	76 (64–82)	72 (60–79)	79 (68–84)	.00
Male, n (%)	204 (56.2)	129 (60.0)	75 (50.7)	.08
Charlson Comorbidity Index, median (IQR)	4 (3–6)	4 (3–5)	5 (4–6)	.11
Septic shock, n (%)	227 (62.5)	107 (49.8)	120 (81.1)	.00
Multi-organ failure, n (%)	159 (43.8)	58 (27.0)	101 (68.2)	.00
SOFA score, median (IQR)	9 (6–11)	7 (5–9)	11 (9–12)	.00
Positive blood culture, n (%)	155 (42.7)	86 (40.0)	69 (46.6)	.21
CRP (mg/dl), median (IQR)	10.35 (3.63–20.19)	9.67 (3.02–18.97)	11.77 (4.89–21.53)	.04
Procalcitonin, median (IQR)	2.69 (0.49–13.20)	2.57 (0.45–14.92)	2.83 (0.54–11.95)	.13
Six-hour lactate, median (IQR)	3.0 (1.9–5.6)	2.3 (1.7–3.7)	4.6 (2.7–7.1)	.00
Lactate clearance, median (IQR)	26.1 (3.4–47.8)	33.3 (11.1–51.6)	15.7 (−6.7–36.6)	.00

### 3.2 Lactate levels versus lactate clearance

**Figure 2:**
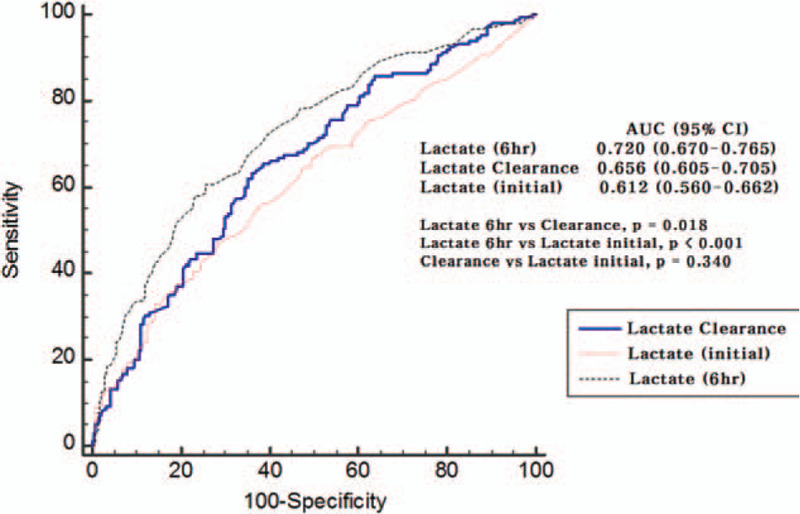
Receiver operating characteristic curves of the 6-hour lactate and initial lactate levels and lactate clearance. AUC = area under the curve, CI = confidence interval.

**Figure 3:**
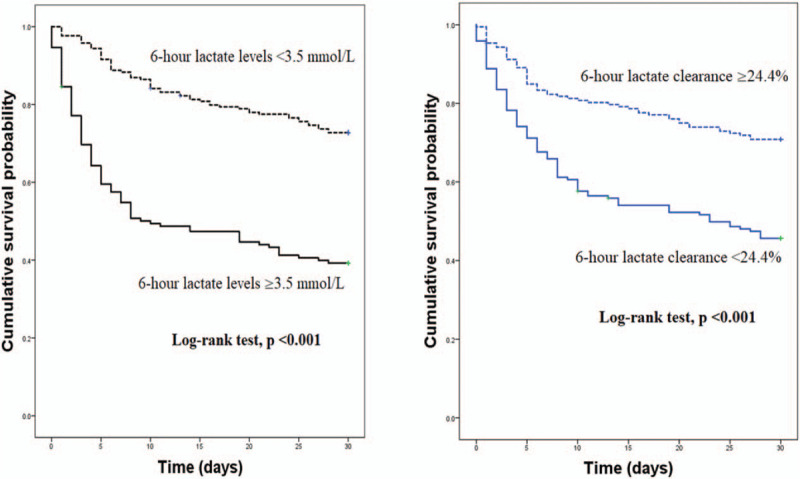
Kaplan–Meier survival curves stratified by the optimal cut-off value of 6-hour lactate levels and lactate clearance to predict 30-day mortality.

Both 6-hour lactate levels and 6-hour lactate clearance were associated with the 30-day mortality after adjusting for confounders (age, the SOFA score and initial lactate level) in a multivariate logistic regression model (OR, 1.148 [95% CI, 1.049–1.255] and 0.992 [95% CI, 0.986–0.997], respectively) (Table 2). The 6-hour lactate level had better prognostic performance than lactate clearance (AUC, 0.720 [95% CI, 0.666–0.773] vs. 0.656 [95% CI, 0.599–0.713]; *P* = .02) and initial lactate level (AUC, 0.720 [95% CI, 0.666–0.773] vs 0.612 [95% CI, 0.522–0.672]; *P* = .00) (Table 3 and Fig. 2). However, the prognostic values of lactate clearance and initial lactate level did not differ significantly (*P* = .34). Six-hour lactate level greater than or equal to 2 mmol/L was associated with 30-day mortality (OR, 1.723 [95% CI, 1.195–3.832]) in the multivariate logistic analysis after adjusting for age, the SOFA score and initial lactate levels. A 6-hour lactate level of ≥3.5 mmol/L was the optimal cut-off value with a sensitivity of 60.8% and specificity of 74.4% in predicting the 30-day mortality. In the Kaplan–Meier survival curve analysis, mortality was significantly higher in patients with higher 6-hour lactate levels (log-rank test, *P* = .00) (Fig. 3). Moreover, all lactate clearance metrics (<10%, <20%, and <30%) were associated with 30-day mortality (OR, 1.823 [95% CI, 1.057–3.712], OR, 1.971 [95% CI, 1.143–3.399], and OR, 1.765 [95% CI, 1.039–3.183], respectively) (Table 2). There was no significant difference between the AUC for each metric (6-hour lactate ≥2 = 0.623 [95% CI, 0.566–0.680]; lactate clearance <10% = 0.605 [95% CI, 0.545–0.665], <20% = 0.626 [95% CI, 0.567–0.685], <30% = 0.619 [95% CI, 0.561–0.678]) (Table 2). A lactate clearance of <24.4% was the optimal cut-off value with a sensitivity of 63.5% and specificity of 63.7% in predicting the 30-day mortality. In the Kaplan–Meier survival curve analysis, mortality was significantly higher in patients with lower lactate clearance (log-rank test, *P* *=* .00) (Fig. 3).

**Table 2:** Logistic regression analysis of 6-hour lactate level and lactate clearance for the prediction of 30-day mortality.

Adjustment	Unadjusted		Adjusted (for age, the SOFA score and initial lactate levels)	
variable	odds ratio	95% CI	odds ratio	95% CI
Lactate (6-hour) (mmol/L)	1.265	1.166–1.373	1.148	1.049–1.255
Lactate (6-hour) ≥2 mmol/L	4.511	2.502–8.132	1.723	1.195–3.832
Lactate clearance	0.989	0.984–0.995	0.992	0.986–0.997
Clearance <10%	2.651	1.679–4.186	1.823	1.057–3.712
Clearance <20%	2.837	1.838–4.379	1.971	1.143–3.399
Clearance <30%	2.682	1.732–4.152	1.765	1.039–3.183

**Table 3:** Prognostic value and test characteristics of lactate levels and lactate clearance for predicting 30-day mortality.

Variable	Area under the curve (95% CI)	Optimal cut-off value	Sensitivity (%) (95% CI)	Specificity (%) (95% CI)	Positive predictive value (%)	Negative predictive value (%)
Lactate clearance (%)	0.656 (0.605–0.705)	<24.4%	63.5	63.7	54.7	71.7
Clearance <10%	0.605 (0.545–0.665)		43.2 (35.1–51.6)	77.7 (71.5–83.1)	57.1	66.5
Clearance <20%	0.626 (0.567–0.685)		56.8 (48.4–64.9)	68.4 (61.7–74.5)	55.3	69.7
Clearance <30%	0.619 (0.561–0.678)		67.6 (59.4–75.0)	56.3 (49.4–63.0)	51.5	71.6
Lactate (initial) (mmol/L)	0.612 (0.560–0.662)	≥7.6 mmol/L	33.1	85.6	61.3	65.0
Lactate (6-hour) (mmol/L)	0.720 (0.670–0.765)	≥3.5 mmol/L	60.8	74.4	62.1	73.4
Lactate (6-hour) ≥2 mmol/L	0.623 (0.566–0.680)		89.2 (83.0–93.7)	35.3 (29.0–42.1)	48.7	82.6

### 3.3 Test characteristics of subsequent lactate levels and lactate clearance metrics

The sensitivity, specificity, positive predictive value, and negative predictive value for each metric are presented in Table 3. We compared the sensitivity and specificity between all metrics using the McNemar test (all *P* values between the metrics = .00). Six-hour lactate levels greater than or equal to 2 mmol/L had the highest sensitivity (89.2% [95% CI, 83.0%–93.7%]) and the lowest specificity (35.3% [95% CI, 29.0%–42.1%]). Lactate clearance of <10% had the highest specificity (77.7% [95% CI, 71.5%–83.1%]) and the lowest sensitivity (43.2% [95% CI, 35.1%–51.6%]).

### 3.4 Association between septic shock/multi-organ failure and mortality

**Figure 4:**
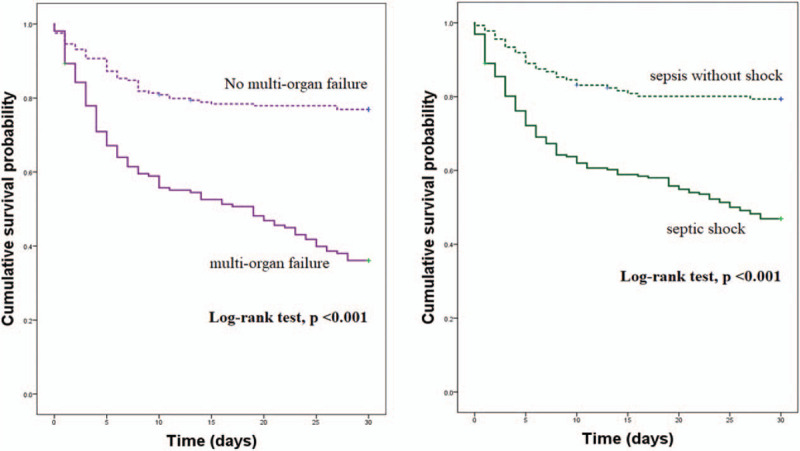
Kaplan–Meier survival curves stratified by the presence of multi-organ failure and septic shock to predict 30-day mortality.

We investigated the association between mortality and septic shock/multiple organ failure using univariable logistic regression analysis. The ORs (95% CI) of septic shock and multi-organ failure was 4.326 (2.649–7.065) and 3.825 (2.347–5.916), respectively (both *P* < .001) In multivariate logistic regression analysis using “backward likelihood ratio (LR) elimination method,” septic shock and multiple organ failure were not risk factors for 30-day mortality. This result is associated with multicollinearity between septic shock/multiple organ failure and lactate metrics. Kaplan–Meier survival curves of multi-organ failure and septic shock were significantly different from those of no multi-organ failure and sepsis without shock (Log-rank test, all *P* < .001) (Fig. 4).

### 3.5 Subgroup analysis and sensitivity analysis

Subgroup analysis according to the disease severity (sepsis vs septic shock) found that 6-hour lactate levels in septic shock group have the highest AUCs and initial lactate levels in sepsis group have the lowest AUCs to predict 30-day mortality (Table 4). Two sensitivity analyses were conducted. First, when we excluded patients who were transferred from other institutions (86 patients excluded from the sensitivity analysis), AUCs (95% CI) of 6-hour lactate levels and lactate clearance were 0.712 (0.651–0.774) and 0.643 (0.585–0.701), respectively. Second, when severe sepsis and septic shock were defined by using the previous Sepsis-2 definitions (12 patients excluded from the sensitivity analysis), AUCs (95% CI) of 6-hour lactate levels and lactate clearance were 0.717 (0.664–0.769) and 0.653 (0.601–0.705), respectively. Both of the sensitivity analyses returned results similar to those of the main analysis.

**Table 4:** Predictive value of each variable for 30-day mortality stratified by the disease severity (sepsis vs septic shock).

	Total (n = 363)	Sepsis (n = 136)	Septic shock (n = 227)
Variable	AUC (95% CI)	AUC (95% CI)	AUC (95% CI)
Lactate clearance (%)	0.656 (0.605–0.705)	0.651 (0.534–0.768)	0.654 (0.583–0.724)
Clearance <10%	0.605 (0.545–0.665)	0.644 (0.522–0.765)	0.592 (0.518–0.665)
Clearance <20%	0.626 (0.567–0.685)	0.647 (0.528–0.765)	0.606 (0.532–0.679)
Clearance <30%	0.619 (0.561–0.678)	0.618 (0.502–0.734)	0.608 (0.534–0.682)
Lactate (initial)	0.612 (0.560–0.662)	0.495 (0.361–0.629)	0.598 (0.524–0.671)
Lactate (6-hour)	0.720 (0.670–0.765)	0.650 (0.530–0.771)	0.697 (0.630–0.765)
Lactate (6-hour) ≥2 mmol/L	0.623 (0.566–0.680)	0.633 (0.524–0.741)	0.585 (0.510–0.660)

## 4 Discussion

In this study, both 6-hour lactate levels and 6-hour lactate clearance showed prognostic value for predicting the 30-day mortality in patients with sepsis and septic shock in accordance with the Sepsis-3 definitions. Nguyen and colleagues defined lactate clearance as the percentage decrease in lactate levels from ED presentation to 6 hours later.^[8]^ They reported that a lactate clearance of <10% was the optimal cut-off value with a sensitivity of 44.7% and specificity of 84.4% in predicting in-hospital mortality. In our study, a lactate clearance of <10% had a sensitivity of 43.2% and specificity of 77.7% in predicting the 30-day mortality, and lactate clearance of <24.4% was the optimal cutoff in predicting the 30-day mortality (sensitivity, 63.5%; specificity, 63.7%).

A recent study concluded that 6-hour lactate levels and lactate clearance were associated with 28-day mortality rates in septic shock patients diagnosed based on the Sepsis-3 definitions.^[16]^ The study also compared the lactate level metrics (≥2, ≥3, and ≥4 mmol/L) and lactate clearance metrics (<10%, <20%, and <30%) to predict the 28-day mortality. Similar to the findings in our study, the previous study also showed that subsequent 6-hour lactate levels ≥2 mmol/L had the highest sensitivity for predicting mortality among the overall metrics. However, the previous study included only septic shock patients (lactate level >2 mmol/L and vasopressor required). Despite the difference in inclusion criteria, the previous study reported that 6-hour lactate levels had better prognostic value than lactate clearance, which is consistent with our results. Furthermore, our study showed that 6-hour lactate levels were superior to initial lactate levels in predicting the 30-day mortality.

Another study compared mortality prediction between subsequent lactate levels (≥4 mmol/L) and lactate clearance (<10% and <20%) in severe sepsis and septic shock patients.^[14]^ According to the study, subsequent lactate levels ≥4 mmol/L and lactate clearance <20% were associated with increased in-hospital mortality, whereas lactate clearance <10% did not increase hospital mortality.^[14]^ In contrast to the findings of that study, our results showed that all lactate clearance metrics (<10%, <20%, and <30%) were associated with 30-day mortality. Definitions (Sepsis-2), inclusion criteria, outcome measure (in-hospital mortality), and measurement time of the subsequent lactate level used in the previous study were different from those in our study, which might be attributable for the difference in results between the 2 studies. In particular, the analysis of the previous study was limited by variations in the time of lactate level measurements.

Serum lactate levels indicate an interaction between the production and elimination of lactate.^[11]^ Previous studies reported that serial lactate measurements are better prognosticators than a single lactate measurement in the shock state.^[21,22]^ A recent study reported that the raw value of a second lactate measurement (AUC = 0.85) within 24 hours had a greater ability to predict short-term mortality than the initial lactate levels (AUC = 0.73) and lactate clearance (AUC = 0.77) in severely injured patients.^[22]^ Given the different conditions, our study also showed that 6-hour lactate levels had a better prognostic value than the initial lactate levels and lactate clearance. A previous study^[23]^ reported that the optimal cut-off values to predict survival are <3.7 mmol/L for the second lactate measurement and ≥32% for lactate clearance. The results of this study were similar to those of our study (≥3.5 mmol/L for 6-hour lactate and <24.4% for lactate clearance to predict 30-day mortality).

We found that the 6-hour lactate level of ≥3.5 mmol/L and initial lactate level of ≥7.6 mmol/L were the optimal cut-off values to predict mortality. Our results showed that initial lactate levels had a poor prognostic value (AUC: 0.612 [95% CI, 0.560–0.662]) when compared with the 6-hour lactate levels. A previous study reported that initial lactate levels had fair prognostic value (AUC 0.70 [95% CI, 0.62–0.79]) with an optimal cut-off value of ≥2.5 mmol/L to predict 28-day mortality among severe sepsis and septic shock patients.^[12]^ This previous study used Sepsis-2 definitions and initial lactate levels in the analysis, which might have caused the differences in results. Among all lactate metrics analyzed in our study, the subsequent 6-hour lactate levels of ≥2 mmol/L showed the highest sensitivity of 89.2% for predicting mortality. Despite the low specificity (35.3%) of 6-hour lactate levels of ≥2 mmol/L, we postulate that the cut-off value of ≥2 mmol/L can be reasonable and practical to predict mortality quickly in sepsis and septic shock patients because of the fatality of the disease.

In accordance with our study, a recent study reported that higher lactate levels and decreased lactate clearance were associated with 7-day and in-hospital mortality among sepsis patients regardless of the presence of shock.^[12]^ Our study included both sepsis (without shock) and septic shock patients diagnosed based on the Sepsis-3 definitions. A retrospective cohort study showed that the optimal cut-off values of initial lactate level and lactate clearance per hour to predict 30-day mortality were 4 mmol/L and 2.5%/h, respectively.^[24]^ A lactate clearance of 2.5% per hour may be estimated to be 14.1% at 6 hours. In our study, we found that the optimal cut-off values of 6-hour lactate level and lactate clearance were 3.5 mmol/L and 24.4%, respectively.

Previous studies showed that initial lactate levels had prognostic value for predicting mortality in septic patients.^[12,15,24,25]^ However, we found that 6-hour lactate levels had better prognostic value than initial lactate levels in sepsis and septic shock patients. Our results suggest that subsequent lactate kevel measurement is essential for lactate-based sepsis management.

Our previous study showed that i-SMS (including qSOFA alert system) improved overall compliance with SSC guideline bundle.^[18]^ Nevertheless, 30-day mortality rate is still high (40.8%). One of our inclusion criteria was initial qSOFA positive (≥2) results. Therefore, all the included patients in our study met initial qSOFA positive criteria. According to the Sepsis-3 definitions, qSOFA criteria have been developed to identify sepsis patients with poor prognosis, but have limited sensitivity for detecting overall sepsis. Therefore, we postulate that using qSOFA positive as inclusion criteria might be associated with high mortality in the present study (selection bias). Other reasons for high mortality might be relatively high proportion of septic shock (62.5%) and older age (median [IQR]; 76 [64–82] years) compared to previous studies.

There were some limitations to this study. First, because our study was conducted in a single tertiary teaching hospital, the generalizability of our results to external populations remains uncertain. Second, this study was a retrospective study limited by characteristics inherent to retrospective analyses and interpretations. Third, the present study included sepsis and septic shock patients with a positive qSOFA score upon arrival to the ED, which might have resulted in a selection bias. Fourth, because there are numerous factors that may alter lactate metabolism, clinicians should be cautious when applying our results to sepsis and septic shock patients.

## 5 Conclusions

This study showed that 6-hour lactate levels and 6-hour lactate clearance were associated with 30-day mortality in sepsis and septic shock patients. In particular, 6-hour lactate levels had better prognostic performance than 6-hour lactate clearance and initial lactate levels. Among the various metrics analyzed, 6-hour lactate levels of ≥ 2 mmol/L had the greatest sensitivity in predicting patient outcomes. Multicenter prospective studies with a larger sample size are needed to confirm our results.

## Acknowledgments

The authors are grateful to researchers Hye-yoon Jung and Min-sook Jung for their contributions to the project.

## Author contributions

**Conceptualization:** Juhyun Song, Seong Geun Lee, Dae Won Park.

**Data curation:** Juhyun Song, Joo Yeong Kim, Jae Hyung Cha.

**Formal analysis:** Juhyun Song, Seong Geun Lee, Jae Hyung Cha.

**Investigation:** Juhyun Song, Sungwoo Moon, Jonghak Park.

**Methodology:** Juhyun Song, Dae Won Park.

**Project administration:** Han-jin Cho, Sungwoo Moon, Dae Won Park.

**Resources:** Juhyun Song, Seong Geun Lee, Dae Won Park, Han-jin Cho.

**Software:** Juhyun Song, Jae Hyung Cha.

**Supervision:** Sungwoo Moon, Dae Won Park.

**Visualization:** Juhyun Song.

**Writing – original draft:** Juhyun Song, Seong Geun Lee.

**Writing – review & editing:** Dae Won Park, Han-jin Cho.

## Supplementary Material

Supplemental Digital Content
