# Supplementary material for: Prognostic value of lactate levels and lactate clearance in sepsis and septic shock with initial hyperlactatemia: A retrospective cohort study according to the Sepsis-3 definitions
Source: Medicine (Baltimore). 2021 Feb 19;100(7):e24835. doi: 10.1097/MD.0000000000024835 (PMC7899836; doi:10.1097/MD.0000000000024835)
Supplement: Supplemental Digital Content [file medi-100-e24835-s001.docx]

**Prognostic value of lactate levels and lactate clearance in sepsis and septic shock with initial hyperlactatemia: a retrospective cohort study according to the Sepsis-3 definitions**

Seong Geun Lee, Juhyun Song, Dae Won Park, Sungwoo Moon, Han-jin Cho, Joo Yeong Kim, Jonghak Park, Jae Hyung Cha

**Supplementary Table 1.** **Baseline characteristics of included patients and excluded patients who lacked 6-hour lactate levels.**

| **Variable** | **Included**  **(n = 363)** | **Excluded**  **(n = 252)** | ***P-*value** |
| --- | --- | --- | --- |
| **Age, median (IQR)** | 76 (64–82) | 75 (62–81) | .24 |
| **Male, n (%)** | 204 (56.2) | 139 (55.2) | .21 |
| **Charlson Comorbidity Index, median (IQR)** | 4 (3–6) | 4 (3–6) | .48 |
| **Septic shock, n (%)** | 227 (62.5) | 151 (59.9) | .34 |
| **Multi-organ failure, n (%)** | 210 (57.9) | 139 (55.2) | .41 |
| **SOFA score, median (IQR)** | 9 (6–11) | 8 (5–11) | .18 |
| **Positive blood culture, n (%)** | 155 (42.7) | 101 (40.1) | .26 |
| **CRP (mg/dL), median (IQR)** | 10.35 (3.63–20.19) | 10.03 (3.24–19.57) | .17 |
| **Procalcitonin, median (IQR)** | 2.69 (0.49–13.20) | 2.58 (0.43–12.59) | .15 |
| **30-day mortality rate, n (%)** | 148 (40.8) | 96 (38.1) | .18 |

CRP, C-reactive protein; IQR, interquartile range
